# Hydroxyurea mitigates diabetic kidney disease through mTOR-S6K signaling pathway in STZ-induced diabetic mice

**DOI:** 10.3389/fcell.2025.1529901

**Published:** 2025-01-22

**Authors:** Wanying Cheng, Cenzhu Wang, Meican Ma, Yu Zhou

**Affiliations:** ^1^ Department of Hematology, The Affiliated Wuxi People’s Hospital of Nanjing Medical University, Wuxi People’s Hospital, Wuxi Medical Center, Nanjing Medical University, Wuxi, China; ^2^ Department of Oncology, The Affiliated Wuxi People’s Hospital of Nanjing Medical University, Wuxi People’s Hospital, Wuxi Medical Center, Nanjing Medical University, Wuxi, China; ^3^ Department of Rheumatology, The Affiliated Wuxi People’s Hospital of Nanjing Medical University, Wuxi People’s Hospital, Wuxi Medical Center, Nanjing Medical University, Wuxi, China; ^4^ Clinical Research Center, The Affiliated Wuxi People’s Hospital of Nanjing Medical University, Wuxi People’s Hospital, Wuxi Medical Center, Nanjing Medical University, Wuxi, China

**Keywords:** diabetic kidney disease, hydroxyurea, inflammation, apoptosis, s6k

## Abstract

**Background:**

Diabetic kidney disease (DKD) is the leading risk factor for end-stage renal disease (ESRD). Hydroxyurea (HU), a sickle cell disease (SCD) drug approved by FDA, shows protective effect in nephropathy. This study aims to understand whether the application of HU could be effective to treat DKD.

**Methods:**

The streptozotocin (STZ)-induced diabetic mice, and high glucose (HG)-treated human renal mesangial cells (HRMCs) were used to investigate the effect of HU on DKD. Serum creatinine and blood urea nitrogen levels reflecting renal function were evaluated. Histology was used to evaluate pathological changes. Indicators of inflammation and apoptosis were detected. Lastly, the mTOR-S6K pathway was explored by detecting the protein expression of S6K and phosphorylated S6K.

**Results:**

In STZ-induced diabetic mice, administration of HU (20 mg/kg) in drinking water for 16 weeks resulted in significant reductions in creatinine and urea nitrogen levels, alongside mitigating histopathological damage. Additionally, HU effectively suppressed the inflammatory response and apoptosis within the kidneys. HRMC cells were cultivated in HG conditions, and HU effectively attenuated the HG-induced inflammation and apoptosis. Moreover, HU treatment significantly inhibited the mTOR signaling pathway in both in both *in vivo* and *in vitro* experiments.

**Conclusion:**

This study unveils a new role of HU in alleviating diabetic kidney disease by modulating inflammation and apoptosis through the mTOR-S6K pathway. However, since HU did not significantly affect blood glucose levels, its therapeutic potential may be best realized when used in combination with standard antidiabetic therapies. Such a combination approach could simultaneously address hyperglycemia and renal dysfunction, offering a more comprehensive management strategy for DKD.

## Introduction

Diabetic kidney disease (DKD) is a common diabetic complication, the incidence of which is about 30% in patients with type 1 diabetes mellitus (T1DM) and about 40% in patients with type 2 diabetes mellitus (T2DM) worldwide (Zhong et al., 2022). It is a major cause of end-stage renal disease (ESRD). In addition, the high cost of dialysis and kidney transplantation increase the economic burden globally (Xiang et al., 2020). Therefore, it is urgent to find new approaches to prevent the development and progression of DKD.

Previous studies have shown inflammation, fibrosis, and apoptosis leading to progressive loss of kidney function in the progression of DKD (Wada and Makino, 2013). Various inflammatory cytokines, including tumor necrosis factor (TNF), interleukin 1 beta (IL-1β), and interleukin 6 (IL-6) are critically involved in inflammatory cell infiltration and interstitial fibrosis in DKD (Xiang et al., 2020). In addition, intercellular cell adhesion molecule-1 (ICAM-1) deficiency significantly reduced the infiltration of macrophages in the glomerulus in diabetic animal models (Wada and Makino, 2013). High concentration of glucose in DKD induces apoptosis of podocytes, glomerular endothelial cells, and so on, leading to tubulointerstitial fibrosis and glomerulosclerosis (Zheng et al., 2021). Thus, inhibiting inflammation could improve the outcome in DKD patients.

Hydroxyurea (HU) is a therapeutic agent approved by FDA to treat sickle cell disease (SCD) (Ware and Dertinger, 2021). Sickle cell nephropathy (SCN) has been recognized as a major cause of mortality in SCD patients. Abnormal albuminuria, a biomarker of glomerular injury, was found in 20.7% of adolescents and 68% of adults with SCD (Bartolucci et al., 2016). Previous studies have shown that HU treatment significantly attenuated albuminuria and serum creatinine in SCN, thereby improving renal function (Taylor et al., 2019). However, the effect and underlying mechanism of HU on DKD remain unclear.

In this study, we postulated that HU could mitigate renal dysfunction in DKD mice by inhibiting inflammation and apoptosis. To test this hypothesis, further experiments were conducted in the kidneys of diabetic mice, as well as in HG-treated HRMC cells. Furthermore, the molecular mechanism underlying HU’s protective effects against DKD was explored.

## Materials and methods

### Animals

8-week-old male C57BL/6J mice were purchased from the Animal Core Facility of Nanjing Medical University, Nanjing, China. The mice were housed in standard laboratory cages, with a maximum of 5 animals per cage, under SPF conditions, with regulation of temperature (22°C ± 1°C) and humidity (55%) and a 12-h:12-h dark-light cycle, and were free to get food and water. All animal experiments were approved by the Institutional Committee for the Care and Use of Animals at Nanjing Medical University (NO. IACUC-2106002) and in accordance with the ethical guidelines published by the International Council for Laboratory Animal Science (ICLAS).

**FIGURE 1:**
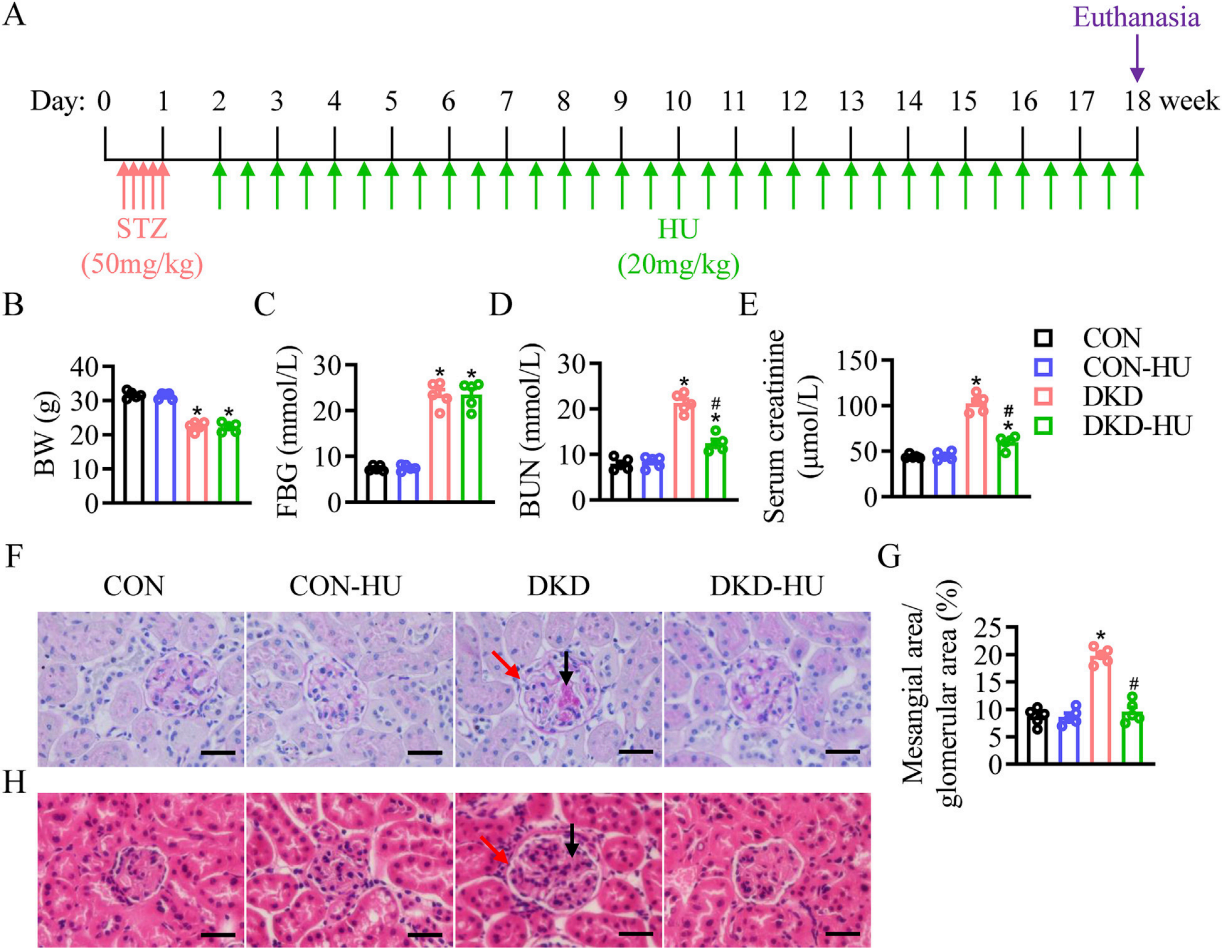
Effects of HU treatment on body weight, blood glucose, renal function and pathological manifestations of renal tissue in STZ-induced DKD mice. **(A)** Schematic representation of the experiment. **(B)** Body weight (BW). **(C)** Fasting blood glucose (FBG). **(D)** Blood urea nitrogen (BUN) level. **(E)** Serum creatinine level. n = 5, *p < 0.05 versus CON; ^#^ *p* < 0.05 versus DKD. **(F)** Representative images of PAS staining. **(G)** The quantification of the mesangial area/glomerular area (%). **(H)** Representative images of HE staining. Black arrows indicate mesangial matrix accumulation and red arrows indicate enlarged glomerular volume. Scale bar = 50 μm.n = 5, *p < 0.05 versus CON; ^#^ *p* < 0.05 versus DKD. CON, Control; DKD, Diabetic kidney disease.

The mice were randomly divided into four groups (n = 5/group): the CON, CON-HU, DKD, and DKD-HU groups. DKD mice was established by intraperitoneal injection of 50 mg/kg streptozotocin (HY-13753; MedChemExpress, China) for 5 days. Control mice were injected with the same volume of citric acid buffer. A week later, blood samples were collected from the tail tips of the mice for blood glucose measurement by a blood glucose meter (FreeStyle Optium Neo; Abbott, United States of America) following a 6-h fasting period. Mice with fasting blood glucose (FBG) ≥16.7 mM were considered diabetic. 20 mg/kg HU (H106352; Aladdin, China) was administered to mice by adding to drinking water for 16 weeks, and was changed to fresh twice a week. The experiment ended when the mice were 26 weeks old. Mice were humanely euthanized via cervical dislocation. The detailed process was shown in Figure 1A.

### Cell culture

The cell line of HRMCs was purchased from the Chinese Academy of Sciences, Shanghai Institute for Biological Sciences Cell Resource Center. HRMCs were cultured in DMEM medium containing 10% fetal bovine serum (FBS), and 1% penicillin/streptomycin. Cells were exposed to either 27.5 mM glucose or 27.5 mM mannitol. HU was then mixed into the medium at the specified concentrations and incubated for 48 h. Cells were collected for further analysis at the end of the experiment. Each experiment was replicated at least three times.

### Analysis of renal function

Blood urea nitrogen (BUN) and creatinine levels were quantitatively measured by BUN Assay Kit (C013-one to one, Njjcbio, China) and Creatinine Assay Kit (C011-2-1, Njjcbio, China) according to manufacturer’s instructions. After reaction with the relevant reagents, the absorbance of BUN was read at 640 nm and the absorbance of serum creatinine was read at 546 nm using a SpectraMax M2e plate reader (Molecular Devices).

### Histology

Kidney tissues were fixed with 4% paraformaldehyde and cut into 4 μm sections after embedded in paraffin. The stained sections were observed with a microscope (BX43, Olympus, Japan). Hematoxylin and eosin (H&E), Periodic acid-schiff (PAS) and Masson staining were used to assess kidney lesions. ImageJ software version 1.52a (Wayne Rasband, National Institutes of Health, United States) was used to quantify the mesangial area and analyze Masson intensity.

### Enzyme-linked immunosorbent assay (ELISA)

Equal amounts of kidneys from each group were homogenized in lysate, and inflammatory cytokines, includingTNF-α, IL-1β, IL-6 were measured in the lysed supernatant using ELISA kits according to the manufacturer’s instructions (Thermo Fisher Scientific, United States).

### Apoptosis assay

Apoptosis of kidney and HRMC cells was detected using a TUNEL FITC Apoptosis Detection Kit (A111; Vazyme, United States). The TUNEL positive nuclei were photographed using a Laser confocal microscope (Axio-Imager_LSM-800, Carl Zeiss, Germany). ImageJ software version 1.52a (Wayne Rasband, National Institutes of Health, United States) was used to analyze TUNEL-positive rate.

### Cell viability assay

Cell viability was tested using Cell Counting Kit-8 (BS350B, Biosharp, China). HRMC cells were seeded in a 96-well plate at a density of 3,000 cells per well and treated with various concentrations of HU. 10 μL CCK-8 solution was added to each well and incubated at 37°C for 30 min according to the manufacturer’s instructions. Absorbance was detected at 450 nm with the SpectraMax M2e plate reader (Molecular Devices). The experiments were conducted in triplicate and repeated independently three times.

### Quantitative real-time PCR

Total RNA from kidneys or HRMC cells was isolated by trizol reagent (R401-01; Vazyme, China). 1 μg RNA was reverse-transcribed into cDNA using RT SuperMix (R323-01; Vazyme, China). The mRNA levels of connective tissue growth factor (Ctgf), transforming growth factor beta 1 (Tgfb1), actin alpha 2 smooth muscle aorta (Acta2), collagen type I alpha 1 (Col1a1), collagen type III alpha 1 (Col3a1), tumor necrosis factor (Tnf), interleukin 1 beta (Il1b), interleukin 6 (Il6), intercellular cell adhesion molecule 1 (Icam1) and vascular cell adhesion molecule 1 (Vcam1) were determined by quantitative real-time PCR under a StepOnePlus™ Real-Time PCR System (Applied Biosystems; Foster City, CA). The primers used are listed in Table 1.

**TABLE 1:** Primers used for RT-PCR.

	Gene	Primer sequences (5′-3′)
Mouse	*Ctgf*	Forward: GCT​GCC​TAC​CGA​CTG​GAA​GA
		Reverse: CTT​AGA​ACA​GGC​GCT​CCA​CTC​T
	*Tgfb1*	Forward: GCA​GTG​GCT​GAA​CCA​AGG​A
		Reverse: GCA​GTG​AGC​GCT​GAA​TCG​A
	*Acta2*	Forward: GAC​GCT​GAA​GTA​TCC​GAT​AGA​ACA
		Reverse: GGC​CAC​ACG​AAG​CTC​GTT​AT
	*Col1a1*	Forward: GAC​TGG​AAG​AGC​GGA​GAG​TAC​TG
		Reverse: CCT​TGA​TGG​CGT​CCA​GGT​T
	*Col3a1*	Forward: GGG​AAT​GGA​GCA​AGA​CAG​TCT​T
		Reverse: TGC​GAT​ATC​TAT​GAT​GGG​TAG​TCT​CA
	*Tnf*	Forward: CAG​CCG​ATG​GGT​TGT​ACC​TT
		Reverse: GGCAGCCTTGTCCCTTGA
	*Il1b*	Forward: AGT​TGA​CGG​ACC​CCA​AAA​GA
		Reverse: GGA​CAG​CCC​AGG​TCA​AAG​G
	*Il6*	Forward: CCA​CGG​CCT​TCC​CTA​CTT​C
		Reverse: TTG​GGA​GTG​GTA​TCC​TCT​GTG​A
	*Icam1*	Forward: GGAGGTGGCGGGAAAGTT
		Reverse: TCC​AGC​CGA​GGA​CCA​TAC​AG
	*Vcam1*	Forward: CTG​CTC​AAG​TGA​TGG​GAT​ACC​A
		Reverse: ATC​GTC​CCT​TTT​TGT​AGA​CAT​GAA​G
	*Gapdh*	Forward: AGA​ACA​TCA​TCC​CTG​CAT​CC
		Reverse: AGTTGCTG TTGAAGTCGC
Human	*TNF*	Forward: CCT​CTC​TCT​AAT​CAG​CCC​TCT​G
		Reverse: GAG​GAC​CTG​GGA​GTA​GAT​GAG
	*IL1B*	Forward: AAG​TAC​CTG​AGC​TCG​CCA​GTG​AAA
		Reverse: TTG​CTG​TAG​TGG​TGG​TCG​GAG​ATT
	*IL6*	Forward: AAA​TTC​GGT​ACA​TCC​TCG​ACG​GCA
		Reverse: AGT​GCC​TCT​TTG​CTG​CTT​TCA​CAC
	*ICAM1*	Forward: TTT​GAC​AGG​CTG​GAG​ATA​GAC​T
		Reverse: TCA​ATG​TGT​AAT​TTA​GCT​CGG​CA
	*VCAM1*	Forward: ATG​CCC​AGA​CAT​CTG​TGT​CC
		Reverse: GGG​GTC​TCT​ATG​CCC​AAC​AA
	*GAPDH*	Forward: GGA​GCG​AGA​TCC​CTC​CAA​AAT
		Reverse: GGC​TGT​TGT​CAT​ACT​TCT​CAT​GG

### Western blot

Protein extraction was performed by homogenizing kidney tissues or HRMC cells in RIPA lysate buffer containing PMSF protease inhibitor. 25 μg protein was loaded into 8%–12% SDS-PAGE gels for electrophoresis and then transferred to polyvinylidene fluoride (PVDF) membranes. Membranes were blocked in 5% nonfat milk, then incubated with primary antibodies overnight followed by secondary antibodies (Proteintech) for 1 h. An enhanced chemiluminescence detection system (Amersham Imager 600, General Electric, United States) was used to detect signals. Primary antibodies were as following: ACTA2 (14395-1-AP, Proteintech), BCL2 (12789-1-AP, Proteintech), BAX (50599-2-lg, Proteintech), ICAM1 (10831-1-AP, Proteintech), TNF (17590-1-AP, Proteintech), IL6 (sc-28343, Santa Cruz), p-S6K (sc-8416, Santa Cruz), S6K (sc-8418, Santa Cruz), Tubulin (66031-1-Ig, Proteintech) and GAPDH (60004-1-lg, Proteintech).

### Molecular docking

The energy-minimum conformation of hydroxyurea for docking was obtained by the conformation search function of Molecular Operating Environment (MOE v2019.0102, Chemical Computing Group ULC, Montreal, QC, Canada). The S6K protein structure data (PDB ID: 3A62) was obtained from the protein database (RCSB Protein Data Bank-RCSB PDB, www.pdb.org), and the protein was pretreated using the Quick-prep function of MOE, including the addition of polar hydrogens and the complementation of missing amino acid residues. The MOE Dock provides a database of dynamically generated conformations, which are then refined using a force-field-based approach. The London dG scoring function and GBVI/WSA dG scoring function based on force field were used in this experiment. The conformation with the lowest binding free energy is chosen as the most reasonable binding mode. The molecular docking results were processed by MOE.

### Statistical analyses

Statistical analyses were conducted using Prism 8 (GraphPad) software. The data were expressed as mean ± SEM. One-way ANOVA was employed for comparisons among multiple groups with normal distributions, while one-way ANOVA with Tukey’s test was used for non-normally distributed data. Statistical significance was considered at p < 0.05 for all analyses.

## Results

### HU attenuated the renal function and the lesions of renal histopathology in STZ-induced diabetic mice

The results showed that body weight in the DKD group was significantly decreased compared with the CON group (Figure 1B), whereas the fasting blood glucose (FBG) levels were significantly increased (Figure 1C). The HU treatment did not restore these two indicators in DKD mice (Figures 1B, C). Mice in the DKD group had significantly higher blood urea nitrogen (BUN) and serum creatinine levels than those in the CON group. However, the HU treatment diminished these parameters of renal dysfunction in DKD mice (Figures 1D, E). As shown in Figures 1F–H, PAS and H&E staining showed mesangial matrix accumulation (black arrows) and enlarged glomerular volume (red arrows) in DKD mice, which were attenuated by HU treatment.

### HU alleviated renal fibrosis, inflammation, and apoptosis in STZ-induced diabetic mice

**FIGURE 2:**
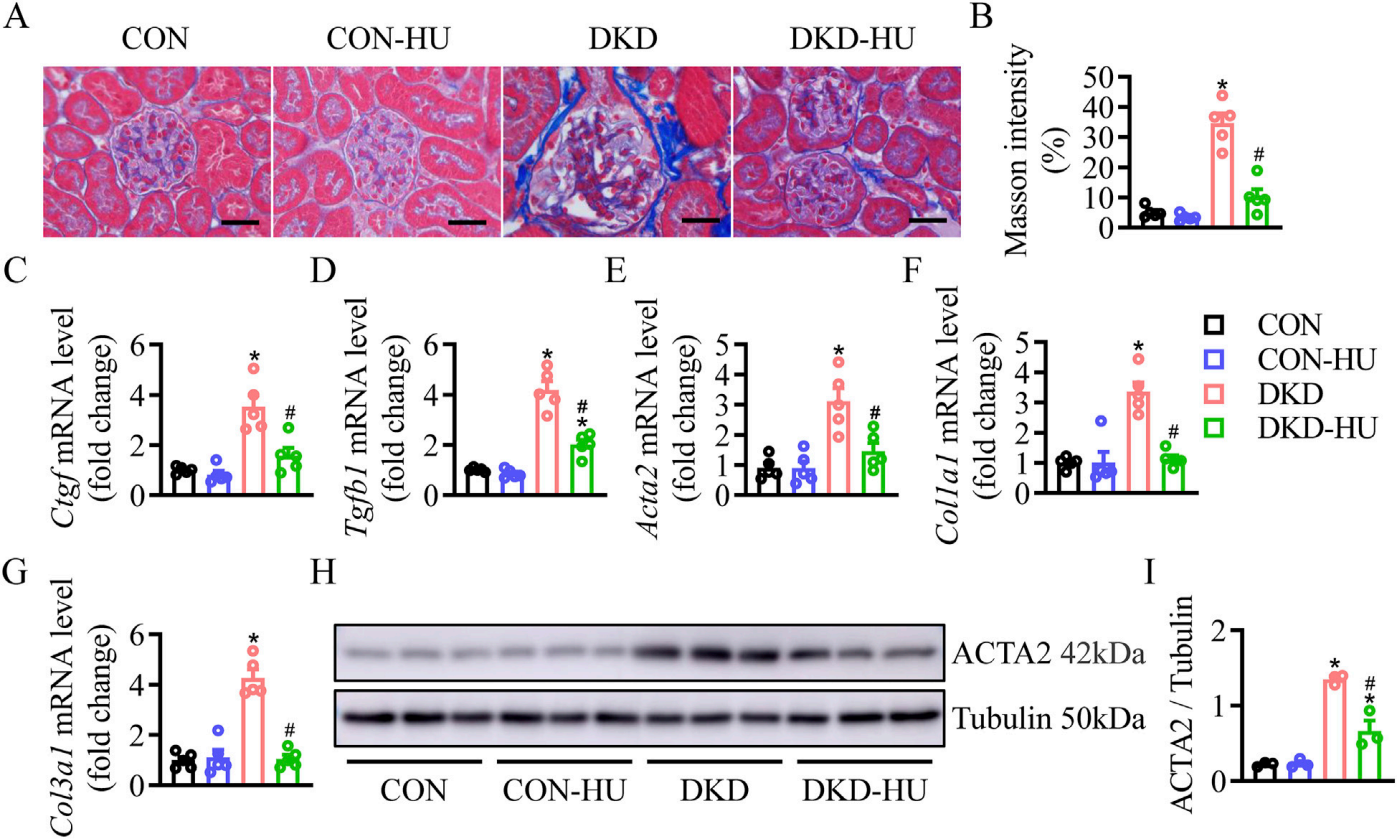
HU alleviated renal fibrosis in STZ-induced diabetic mice. **(A)** Representative images of Masson staining. Scale bar = 50 μm. **(B)** The quantification of Masson staining. **(C–G)** Relative mRNA levels of *Ctgf, Tgfb1, Acta2, Col1a1, and Col3a1*. **(H, I)** Representative blot and quantitative analysis of α-SMA. n = 3–5, *p < 0.05 versus CON; ^#^ *p* < 0.05 versus DKD. CON, Control; DKD, Diabetic kidney disease.

Masson staining showed that HU significantly reduced the aggravated renal fibrosis (stained with blue) in the DKD group (Figures 2A, B). Consistently, the mRNA expressions of *Ctgf*, *Tgfb1*, *Acta2*, *Col1a1*, and *Col3a1* were elevated in the DKD group, but significantly decreased after HU treatment (Figures 2C–G). In consistence with results of mRNA level, HU treatment significantly inhibited the upregulated protein level of α-SMA in the DKD group (Figures 2H, I). Together, these results proved that HU alleviated renal fibrosis in DKD mice.

**FIGURE 3:**
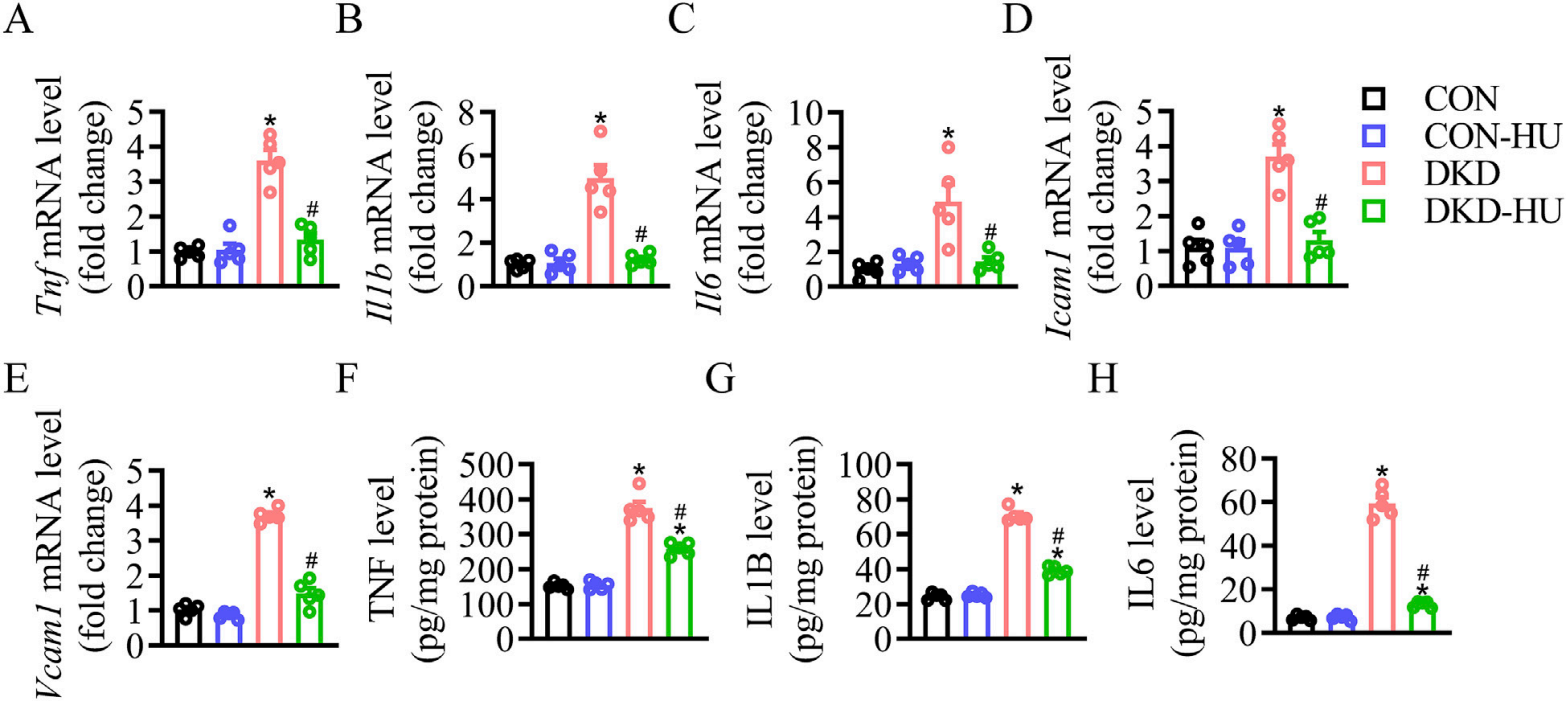
HU reduced renal inflammation in STZ-induced diabetic mice. **(A–E)** Relative mRNA levels of *Tnf*, *Il1b*, *Il6*, *Icam1*, and *Vcam1*. **(F–H)** The protein levels of TNF, IL1B, and IL6 measured by ELISA. n = 5, *p < 0.05 versus CON; ^#^ *p* < 0.05 versus DKD. CON, Control; DKD, Diabetic kidney disease.

Other detrimental markers of inflammation were studied to confirm the efficacy of HU. The mRNA levels of inflammatory factors and cell adhesion factors were increased in the DKD group compared with the CON group, including *Tnf*, *Il1b*, *Il6*, *Icam1* and *Vcam1*, but significantly decreased after HU treatment (Figures 3A–E). The anti-inflammatory effect of HU was further demonstrated by ELISA results (Figures 3F–H).

**FIGURE 4:**
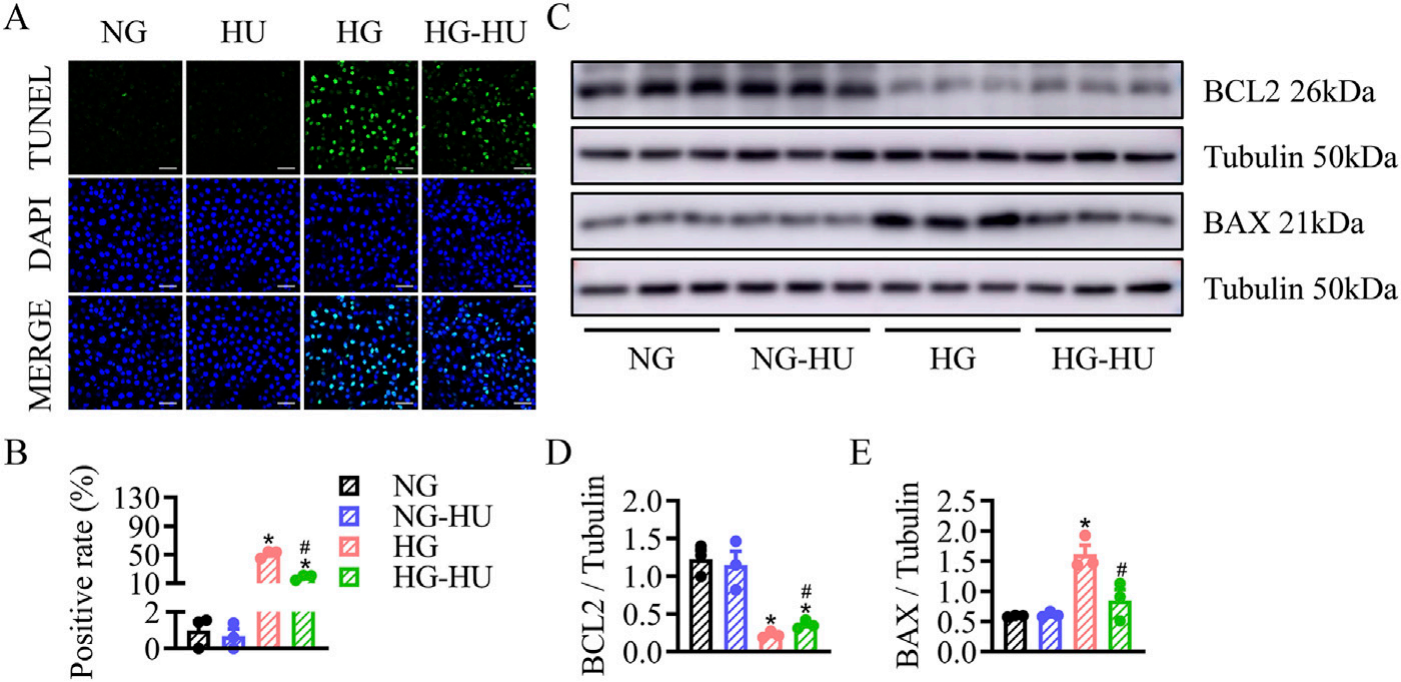
HU relieved renal cell apoptosis in STZ-induced diabetic mice. **(A)** Representative images of TUNEL staining of kidney tissues. Scale bar = 100 μm. **(B)** The quantification of TUNEL-positive cells. **(C–E)** Representative blots and quantitative analysis of BCL2 and BAX. n = 3–5, *p < 0.05 versus CON; ^#^ *p* < 0.05 versus DKD. CON, Control; DKD, Diabetic kidney disease.

Whether HU has anti-apoptotic effect on diabetic kidneys was determined by TUNEL assay. As shown in Figures 4A, B, the increased TUNEL-positive cells in the DKD group were significantly reduced by HU. Compared with the CON group, the BAX protein level was increased, but the BCL2 protein level was decreased in the DKD group, and HU significantly reversed these changes (Figures 4C–E). These data suggest that HU could ameliorate diabetes-induced renal cell apoptosis.

### HU reduced inflammation and apoptosis in HG-treated HRMC cells

**FIGURE 5:**
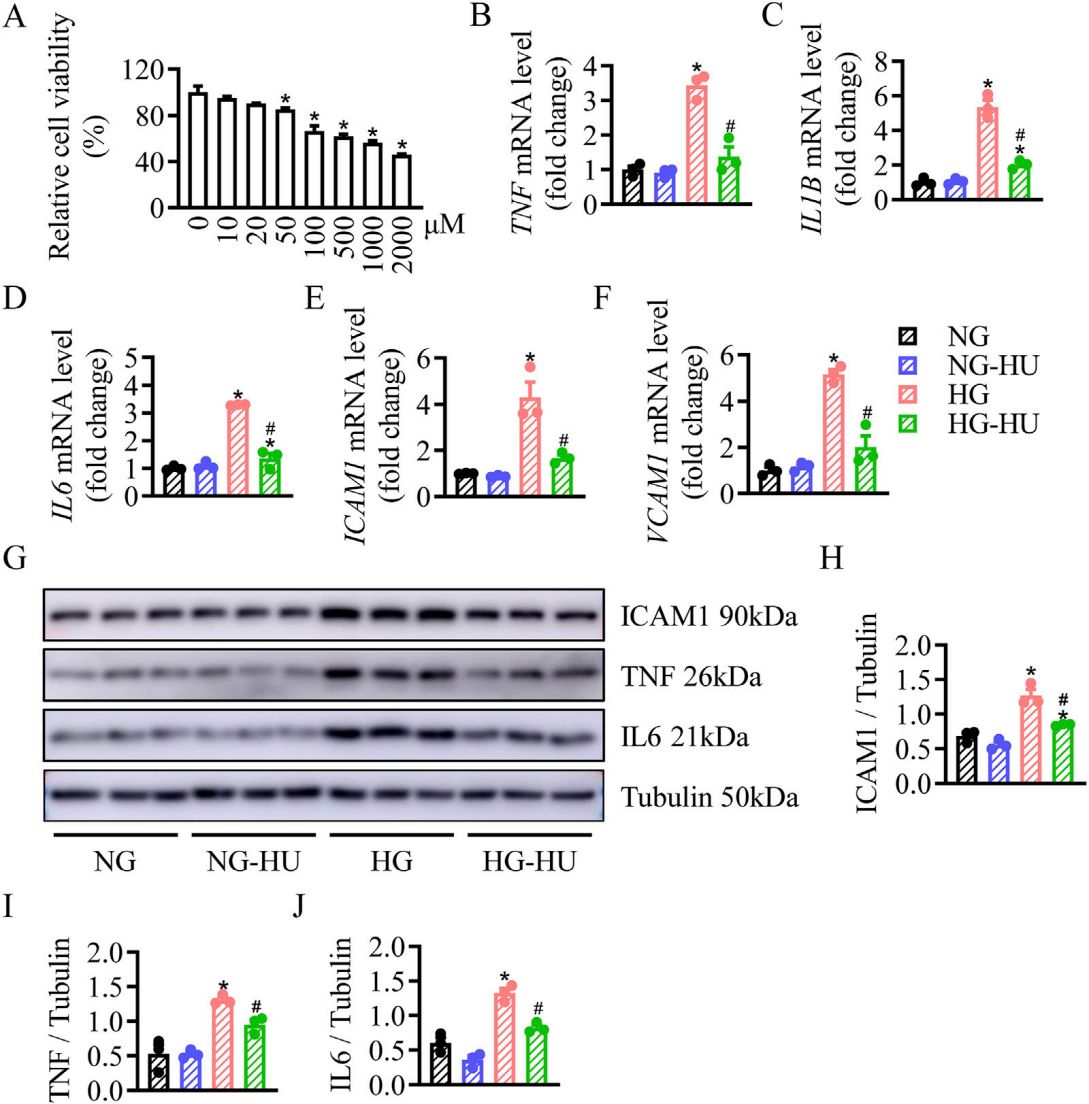
HU reduced HG-induced inflammation in HRMC cells. **(A)** HRMC cells were treated with various concentrations of HU (0–2000 μM) for 48 h, the cell viability was then measured by CCK-8. n = 4, *p < 0.05 versus 0 μM. **(B–F)** Relative mRNA levels of *TNF*, *IL1B*, *IL6*, *ICAM1*, and *VCAM1*. **(G–J)** Representative blots and quantitative analysis of ICAM1, TNF and IL6. n = 3, *p < 0.05 versus NG; ^#^ *p* < 0.05 versus HG. NG, Normal glucose; HG, High glucose.

The changes in cell viability were assessed to determine the proper dosage of HU on HRMC cells. When the concentration was greater than 50 μM, the survival rate of HRMC cells was significantly decreased regarding to high-dose toxicity. Therefore, the minimum dose (10 µM) was chosen as the effective safe concentration of HU treatment (Figure 5A). HU treatment obviously decreased the HG-induced elevated mRNA levels of *TNF*, *IL1b*, *IL6*, *ICAM1*, and *VCAM1* in HRMC cells (Figures 5B–F). Likewise, Western blotting results showed that ICAM1, TNF, and IL6 protein levels were increased in the HG group compared with the NG group, which were inhibited by HU treatment (Figures 5G–J). These data showed anti-inflammatory effects of HU *in vitro*.

**FIGURE 6:**
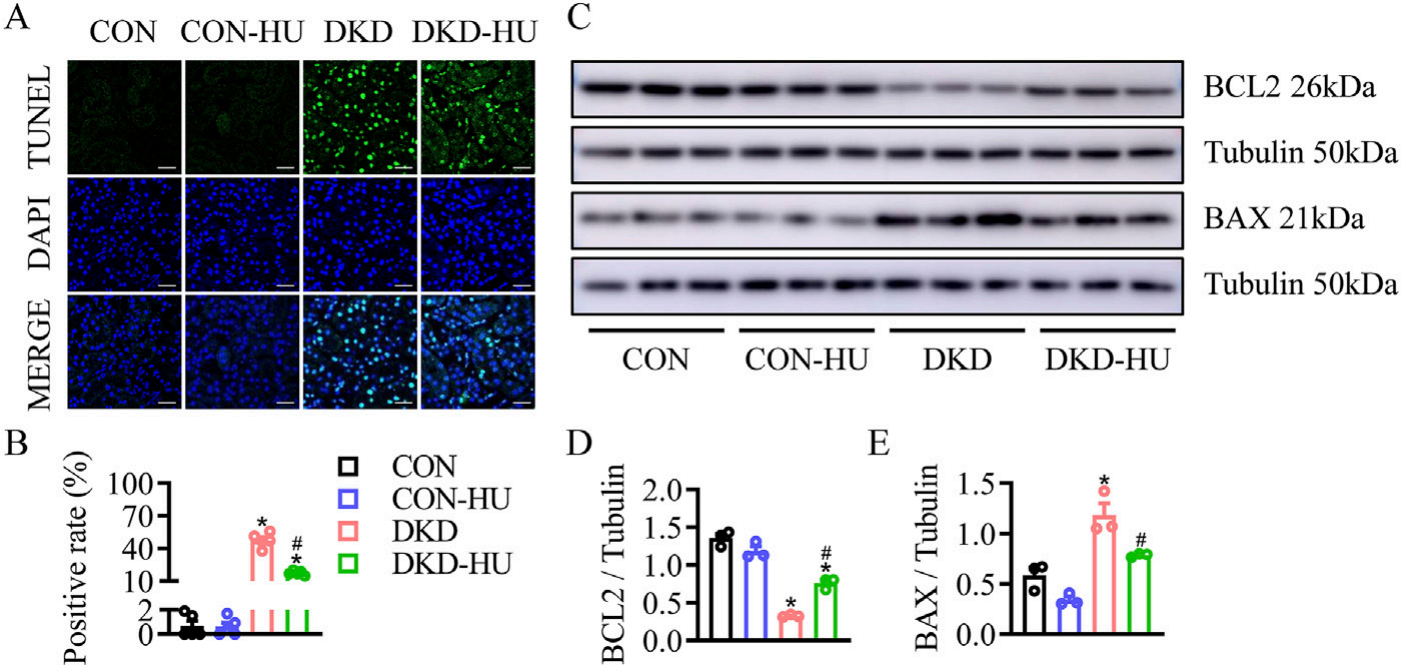
HU relieved HG-induced apoptosis in HRMC cells. **(A)** Representative images of TUNEL staining of HRMC cells. Scale bar = 100 μm. **(B)** The quantification of TUNEL-positive cells. **(C–E)** Representative blots and quantitative analysis of BCL2 and BAX. n = 3, *p < 0.05 versus NG; ^#^p < 0.05 versus HG. NG, Normal glucose; HG, High glucose.

TUNEL staining showed that HU treatment prevented HG-induced apoptosis in HRMC cells (Figures 6A, B). Additionally, Western blot results showed the levels of apoptosis associated proteins including BAX and BCL2. HU treatment significantly recovered the increase of BAX and downregulation of BCL2 induced by HG (Figures 6C–E). These indicated that HU plays a protective role in HRMC cells by reducing apoptosis under HG condition.

### HU inhibited diabetes/HG-induced activation of mTOR signaling pathway

**FIGURE 7:**
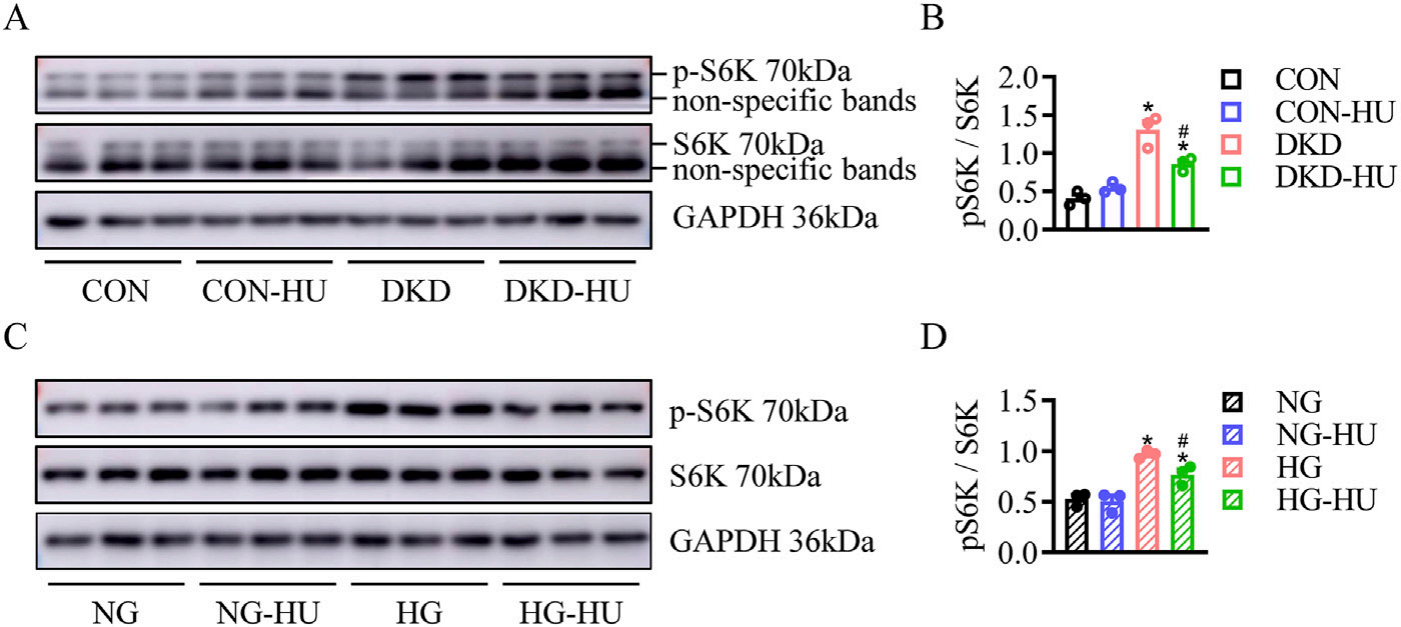
HU inhibited diabetes/HG-induced activation of mTOR signaling pathway. **(A, B)** Representative blots and quantitative analysis of p-S6K in DKD mice. n = 3, *p < 0.05 versus CON; ^#^ *p* < 0.05 versus DKD. CON: Control, DKD: Diabetic kidney disease. **(C, D)** Representative blots and quantitative analysis of p-S6K in HG-treated HRMC cells. n = 3, *p < 0.05 versus NG; ^#^ *p* < 0.05 versus HG. NG: Normal glucose, HG: High glucose.

**FIGURE 8:**
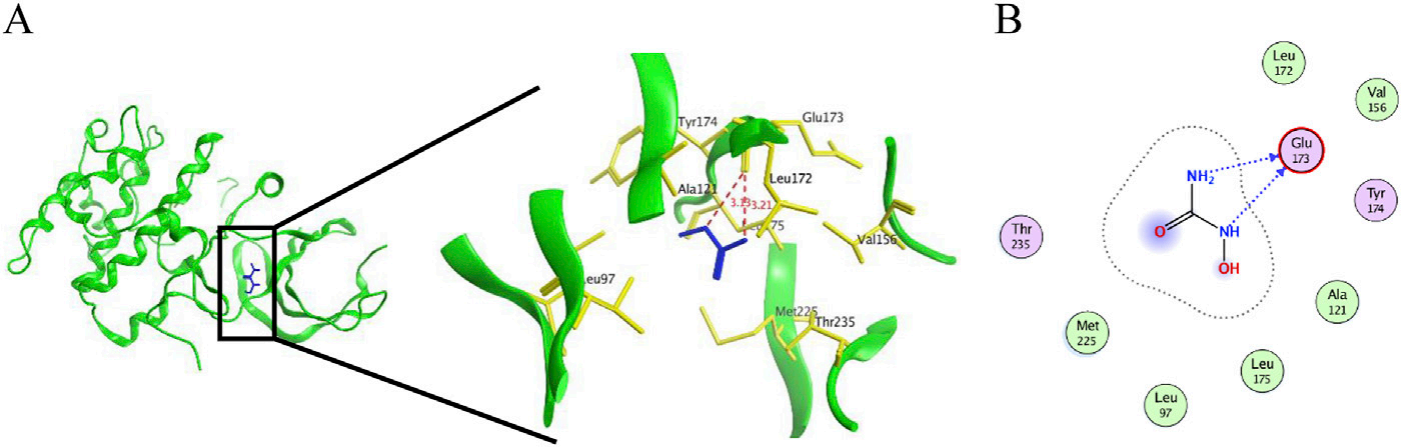
Molecular docking diagram of HU and S6K. **(A, B)** 3D and 2D interaction diagram between S6K and HU.

The mTOR signaling pathway was known to play an important role in regulating autophagy, cell proliferation, metabolism, and oxidative stress (Cheng et al., 2019). Here, the levels of pS6K, a downstream target of mTORC1, were significantly higher in DKD group than in CON group, and the elevated pS6K levels were significantly inhibited after HU treatment (Figures 7A, B). Similarly, pS6K levels were significantly upregulated in HG-treated HRMC cells and improved by HU treatment (Figures 7C, D). Docking studies are performed on MOE to explore the interaction mechanism between HU and S6K. As illustrated in Figures 8A, B, HU forms two hydrogen bonds with Glu173 of S6K with the length of 3.13 Å and 3.21 Å, respectively. Furthermore, HU shows strong hydrophobic interaction with the surrounding amino acids of S6K. These results suggest that HU attenuates kidney injury in STZ-induced diabetic mice by inhibiting the abnormally activated mTOR signaling pathway.

## Discussion

DKD has become the leading cause of end-stage renal disease (ESRD) due to the rapid increase in the incidence of diabetes (Huang et al., 2019). Several approaches, including controlling hyperglycemia and hypertension, reducing obesity, and changing unhealthy lifestyle, are insufficient to retard the progression of DKD. Therefore, more effective interventions to prevent the development of DKD are urgently needed.

HU was approved as an anticancer drug by FDA in 1967 and is currently used to treat conditions such as sickle cell anemia, HIV, and more. Several studies have shown that HU reduces proinflammatory cytokines and inhibits inflammatory processes in rats with myocardial infarction (AMI) or sepsis (Kulturoglu et al., 2022). Ofelia Alvarez et al. found that after 24 months of HU treatment in children with SCA, urine concentration capacity was enhanced and kidney enlargement was reduced, suggesting HU good for renal function (Alvarez et al., 2012). In this study, HU was found to improve renal function by reducing serum creatinine and blood urea nitrogen in diabetic mice. The results of H&E, PAS, and Masson staining showed that HU improved renal histopathology in diabetic mice. HU treatment decreased fibrosis markers in DKD mice.

DKD is generally known as a chronic inflammatory disease. Long-term micro-inflammation results in structure changes of the glomeruli and tubules, finally proteinuria. Studies have shown that hyperglycemia activated the production of inflammatory cytokines such as TNF, IL6, and IL1B, which contribute to kidney damage (Liu et al., 2020). Elevated plasma IL6 levels and persistently upregulated proteinuria in DKD patients accelerate the progression of DKD (Guo et al., 2020). Here, the HU treated STZ-induced diabetic mice and significantly suppressed these inflammatory markers. The results of *in vitro* experiments demonstrated the anti-inflammatory activity of HU against HG-induced cell damage. We found that the expression of inflammatory proteins (TNF and IL6) in HG-treated HRMC cells was significantly reduced by HU treatment. However, no urine from mice was collected in this study. Future studies should test whether HU can reduce proteinuria in DKD.

Apoptosis of tubular epithelial cells impairs normal tubular function, induces tubular atrophy, and promotes the progression of DKD (Huang et al., 2019). HU selectively blocks DNA synthesis and induces cell cycle synchronization or cell death in S phase. On the other hand, the inhibition of DNA replication by HU is reversible. It has been reported that concentrations up to 0.2 mm HU have little effect on the viability or proliferation of NSCLC cells (Oo et al., 2019). This suggests that the cytotoxic effect of HU is dose- and duration-dependent of exposure. Secondly, unlike highly proliferative tumor cells, normal cells are resistant to HU treatment and retain proliferative potential. Previous reports revealed a protective effect of HU on hippocampal neuronal cell viability (Brose et al., 2018), and HU minimized cell death by downregulating the expression level of pro-apoptosis-associated protein (BAX) (Dong et al., 2014). BCL-2 family proteins play a key role in determining cell survival or death through complex interactions between anti-apoptotic and pro-apoptotic proteins. We found that HU played a critical role in alleviating apoptosis in diabetic mice by regulating BAX and BCL2. BAX appears to be inhibited by all anti-apoptotic proteins. Consistent with the *in vivo* results, HU inhibited BAX by upregulating BCL2 expression levels in HG-treated HRMC cells, thereby attenuating apoptosis.

Accumulating evidence suggests that mTOR/S6K signaling is involved in diabetes, cancer, and obesity (Tavares et al., 2015). S6K proteins are representative downstream effectors of mTOR pathway (Wei et al., 2020). Sung Hee Um et al. reported that S6K1 activity was significantly increased in 3 kinds of obese mice model, while S6K1-deficient mice were protected from obesity due to enhanced β-oxidation and showed enhanced insulin sensitivity despite long-term high-fat diets (Um et al., 2004). In the present study, mTOR/S6K activation was observed in kidney tissues of diabetic mice and HG-treated HRMC cells, as indicated by the high phosphorylation of S6K with Western blotting, which was effectively inhibited by HU treatment.

Taken together, our study uncovered that HU ameliorated diabetes-induced kidney damage by inhibiting inflammation and apoptosis. These data provided novel evidence that HU might be an effective and safe drug for DKD. Future research may investigate additional potential applications of HU in the context of inflammation and apoptosis-related diseases.

## Conclusion

**FIGURE 9:**
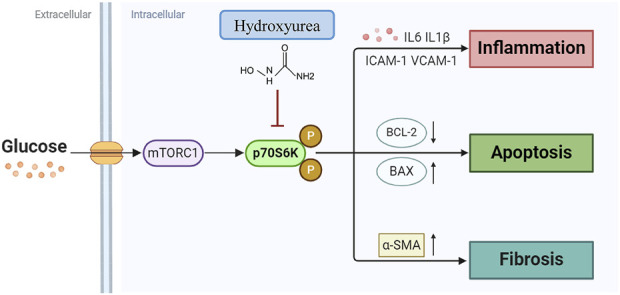
Summary of HU protecting against DKD in this study. Hydroxyurea inhibits inflammation and apoptosis via mTOR-S6K pathway in diabetic kidney disease.

In sum, this study unveils a new role of HU in alleviating diabetic kidney disease by modulating inflammation and apoptosis through the mTOR-S6K pathway (Figure 9). However, since HU did not significantly affect blood glucose levels, its therapeutic potential may be best realized when used in combination with standard antidiabetic therapies. Such a combination approach could simultaneously address hyperglycemia and renal dysfunction, offering a more comprehensive management strategy for DKD. Future studies should explore the synergistic effects of HU with existing glucose-lowering agents to optimize clinical outcomes in patients with DKD.

## Data Availability

The original contributions presented in the study are included in the article/supplementary material, further inquiries can be directed to the corresponding author.

## Abbreviations

*Acta2:* Actin alpha 2; BUN, Blood urea nitrogen; CCK*-8*, Cell counting kit-8; *Col1a1*, Collagen type I alpha 1; *Col3a1*, Collagen type III alpha 1; *Ctgf*, Connective tissue growth factor; DKD, Diabetic kidney disease; ESRD, End-stage renal disease; FBG, Fasting blood glucose; H&E, Hematoxylin and eosin; HG, High glucose; HRMCs, Human renal mesangial cells; HU, Hydroxyurea; *ICAM-1*, Intercellular cell adhesion molecule-1; *IL1β*, Interleukin 1 beta; IL6, Interleukin 6; NG, Normal glucose; PAS, Periodic Acid-Schiff; PVDF, polyvinylidene fluoride; SCD, Sickle cell disease; T1DM, Type 1 diabetes mellitus; T2DM, Type 2 diabetes mellitus; *Tgfb1*, Transforming growth factor beta 1; TNF, Tumor necrosis factor; VCAM*-1*; Vascular cell adhesion molecule-1.
